# Hand Book for Hospital Visitors

**Published:** 1877-08

**Authors:** 


*Hand Book for Hospital Visitors.* New York: G. P. Putnam's Sons, 1877. Buffalo: Peter Paul & Bro.

This is number thirteen of the publications by the STATE CHARITIES AID ASSOCIATION. It is divided into eleven parts, treating upon the various topics naturally most important in the formation of hospital buildings, and their maintenance. It is well written, and will be found to be of much help to managers of hospitals. It embodies all the latest discoveries in ventilation, water supply and methods of warming, together with good advice on the nursing service, care of the insane, and a short chapter on the needs of the maternity ward. Every person having any connection with a hospital should secure this small work, and carefully peruse it. The portion which will be found to be of most practical importance is the appendix. Rules are here given for the government of the nurses and patients, with a diet table, and the quantities necessary for each person. These alone are worth the price of the book. C.

